# *De novo* variants in *MAST4* related to neurodevelopmental disorders with developmental delay and infantile spasms: Genotype-phenotype association

**DOI:** 10.3389/fnmol.2023.1097553

**Published:** 2023-02-22

**Authors:** Xi Zhang, Neng Xiao, Yang Cao, Ying Peng, Aojie Lian, Yuanlu Chen, Pengchao Wang, Weiyue Gu, Bo Xiao, Jing Yu, Hua Wang, Li Shu

**Affiliations:** ^1^Department of Neurology, Xiangya Hospital, Central South University, Changsha, China; ^2^Department of Pediatric Neurology, Chenzhou First People’s Hospital, Chenzhou, China; ^3^Department of Radiology, Chenzhou First People’s Hospital, Chenzhou, China; ^4^National Health Commission Key Laboratory for Birth Defect Research and Prevention, Hunan Provincial Maternal and Child Health Care Hospital, Changsha, China; ^5^Clinical Research Center for Placental Medicine in Hunan Province, Hunan Provincial Maternal and Child Health Care Hospital, Changsha, China; ^6^Department of Pharmacy, Chenzhou First People’s Hospital, Chenzhou, China; ^7^Chigene (Beijing) Translational Medical Research Center Co., Ltd., Beijing, China; ^8^Department of Neurology, Children’s Hospital of Xinjiang Uygur Autonomous Region, Ürümqi, China; ^9^Department of Medical Genetics, Hunan Children’s Hospital, Changsha, China; ^10^Department of Biochemistry, Molecular Biology and Medical Genetics, Cumming School of Medicine, University of Calgary, Calgary, AB, Canada; ^11^Alberta Children’s Hospital Research Institute, University of Calgary, Calgary, AB, Canada

**Keywords:** *MAST4*, neurodevelopmental delay, infantile spasms, genetic, epilepsy

## Abstract

**Objective:**

This study aims to prove that the *de novo* variants in *MAST4* gene are associated with neurodevelopmental disorders (NDD) with developmental delay (DD) and infantile spasm (IS) and to determine the genotype-phenotype correlations.

**Methods:**

Trio-based exome sequencing (ES) was performed on the four families enrolled in this study. We collected and systematically reviewed the four probands’ clinical data, magnetic resonance images (MRI), and electroencephalography (EEG). We also carried out bioinformatics analysis by integrating published exome/genome sequencing data and human brain transcriptomic data.

**Results:**

We described four patients whose median age of seizure onset was 5 months. The primary manifestation was infantile spasms with typical hypsarrhythmia on EEG. Developmental delays or intellectual disabilities varied among the four individuals. Three *de novo* missense variants in *MAST4* gene were identified from four families, including chr5:66438324 (c.2693T > C: p.Ile898Thr) z, chr5:66459419 (c.4412C > T: p.Thr1471Ile), and chr5:66462662 (c.7655C > G:p.Ser2552Trp). The missense variant p.Ile898Thr is mapped to the AGC-kinase C-terminal with phosphatase activity. The other variant p.Ser2552Trp is located in a phosphoserine-modified residue which may affect cell membrane stability and signal transduction. Besides, the variant p.Thr1471Ile is a recurrent site screened out in two unrelated patients. Compared to private mutations (found only in a single family or a small population) of *MAST4* in the gnomAD non-neuro subset, all *de novo* variants were predicted to be damaging or probably damaging through different bioinformatic analyses. Significantly higher CADD scores of the variant p.Thr1471Ile indicate more deleteriousness of the recurrent site. And the affected amino acids are highly conserved across multiple species. According to the Brainspan Atlas database, *MAST4* is expressed primarily in the mediodorsal nucleus of the thalamus and medial prefrontal cortex during the prenatal period, potentially contributing to embryonic brain development.

**Conclusion:**

Our results revealed that the variants of *MAST4* gene might lead to neurodevelopmental disorders with developmental delay and infantile spasm. Thus, *MAST4* variants should be considered the potential candidate gene in patients with neurodevelopmental disorders clinically marked by infantile spasms.

## Introduction

Neurodevelopmental disorders (NDDs) are a group of conditions with a high level of clinical and etiologic heterogeneity, such as attention deficit hyperactivity disorder (ADHD), autism spectrum disorder (ASD), intellectual disability, and learning and communication disorders (Thapar et al., 2017). More than 3% of the population suffers from NDDs, ranging from mild impairments living a fairly normal life to severe disorders requiring lifelong care (Bitta et al., 2017; Mitani et al., 2021). To date, *de novo* variants currently account for only about 25% of unsolved NDD cases (Deciphering Developmental Disorders Study, 2017), indicating more NDD-associated genes remain to be discovered.

*MAST* family members (*MAST1–4*, and *MAST-like*) sharing approximately 49–64% sequence homology contain four distinct domains: The DUF 1,908 domain, the serine/threonine kinase domain, an AGC-kinase C-terminal domain and the PDZ domain (Garland et al., 2008). The *MAST* family plays a critical role in regulating microtubule system and normal cell division in mammals and fruit flies (Chudinova et al., 2017). So far, only *MAST1* and *MAST3* associated with the neurodevelopmental disease have been reported. *MAST1* is highly expressed in the post-mitotic neurons of the cerebellum, cerebral cortex, and hippocampus (Garland et al., 2008; Tripathy et al., 2018). Latterly, *de novo* variants in *MAST1* gene have been linked to mega-corpus-callosum syndrome and intellectual disability (Tripathy et al., 2018; Ben-Mahmoud et al., 2020; Hecher et al., 2020; Rodríguez-García et al., 2020). As a member of the *MAST* family, *MAST3* has recently drawn growing attention as a novel pathogenic gene in neurodevelopmental disorders. More recently, patients with *de novo* variants in *MAST3* are related to neurodevelopmental disabilities with or without epileptic seizure (Iwama et al., 2019; Shu et al., 2021; Spinelli et al., 2021). However, there have been no *MAST2/MAST-L*-related neurological diseases reported. *MAST2* was reported to interact with the PTEN protein, which functions as a negative regulator of cell survival pathways and neuronal survival (Terrien et al., 2012). *MAST-L* is predominantly expressed in only heart and testis rather than in the brain, which is involved in the modulation of the microtubule system (Chudinova et al., 2017).

However, there are only a few reports on the association between *MAST4* and neurological diseases, and the genotype-phenotype correlation needs to be clarified. Genome-wide association studies related *MAST4* variations to myoclonic epilepsy and abnormalities in hippocampal anatomy (EPICURE Consortium et al., 2012; Hibar et al., 2017). Other studies have also reported the association of *MAST4* with other systemic conditions. Furthermore, Mast4(−/−) mice exhibited excessive cartilage synthesis and osteopetrosis phenotype (Kim et al., 2022). In addition, Lee et al. (2021) revealed that *MAST4* is associated with the fibroblast growth factor 2 (FGF2)/ERM pathway and is essential for spermatogenesis in mice.

Given that many other genes of the *MAST* family are highly correlated with neurological disorders, we hypothesized that MAST4 is also associated with it. In this study, we firstly reported three *de novo* missense variants in *MAST4* gene in four unrelated individuals manifesting NDD with DD and epileptic spasms using trio-based ES. The four affected individuals’ clinical data and imaging investigation were collected and systematically reviewed. Together with the bioinformatic analysis, our results support the possibility that *MAST4* relates to neurodevelopmental disorders with infantile spasms as a promising novel pathogenic gene. In addition, we further prove that the *MAST* family members have a critical role in nervous system development.

## Materials and methods

### Study participants

The patients were recruited from an NDD cohort of more than 1,000 at Hunan Provincial Maternal and Child Health Care Hospital. Patients diagnosed with NDD without any identifiable risk factors were enrolled in the study. This study was approved by the Ethics Committee (2020-S003) of Hunan Maternal and Child Health Hospital. The clinical data, laboratory findings, brain MRI and EEG of the four affected individuals were collected and systematically reviewed by experienced neurophysiologists and neuroradiologists. Written informed consent was obtained from all participants in the study.

### Trio-exome sequencing and quality control

The peripheral blood was collected from the probands, their parents and other available family members to determine the origin of the identified genetic variants. Genomic DNA (>3 μg) was extracted using the DNA mini kit (Aidlab Biotech, China). The kinship of four trios’ samples have been checked, and the data is shown in Supplementary Table 1 and novel variants identified in this study were submitted to ClinVar (SCV002817420- SCV002817422). Sequencing reads were aligned to the reference human genome (GRCh37/hg19) using the BWA2 (v0.7.15). Picard was applied to sort by chromosome coordinates and mark duplicates^1^. Single nucleotide variants and insertions/deletions of samples were called and the Variant Call Format (VCF) file was generated (format v4.1) by HaplotypeCaller v4.0 from Genome Analysis Toolkit (GATK) (Van der Auwera et al., 2013). BCFtools (Narasimhan et al., 2016), VCFtools (Danecek et al., 2011) and GATK (v4.1.8.1) were applied to left-align, normalize, extract and retain rare events allele frequency <0.1% in gnomAD v2.1.1 non-neuro subset exomes (Karczewski et al., 2020) in the VCF files. We removed variants within mean mapping quality of mapped reads (MQ) <35, quality by depth (QD) ≤2, Fisher Strand (FS) ≤60, Strand Odds Ratio (SOR) >3, MQ Rank Sum −12.5 and Read Pos Rank Sum ≤−8. Genotype quality (GQ), read-depth (RD) and allele balance (AB) were used for genotype-level quality controls. Variants within 0.25 < AB < 0.75, DP > 10, and GQ > 25 were reserved. Finally, we chose loss of function mutations and missense variants with CADD > 20. Pubvar variant annotation engine and VEP6 (release 88) were used to annotate variants^2^. Relatedness was calculated between each pair of samples using King v2.2.7 (Manichaikul et al., 2010). The pathogenicity of variants was interpreted according to American College of Medical Genetics (ACMG)/AMP guidelines and modified based on ClinGen recommendations. Validation of the identified variants was performed using sanger sequencing.

### Bioinformatic analysis

#### *In silico* variants analysis

To evaluate the conservation of the affected amino acid, we performed multiple sequence alignments across different species (Madeira et al., 2022). The conservation and pathogenicity of missense variations were evaluated through an integrated and accessible genetic database containing multiple prediction tools (i.e., SIFT, REVEL, CADD, and VEST3 et al.) (Li et al., 2018; Kopanos et al., 2019). ANNOVAR was used to annotate all *MAST4* variants (Wang et al., 2010). Published *MAST4 de novo* mutations were obtained from Gene4Denovo^3^. Protein domain structures were visualized using DOG 2.0 (Ren et al., 2009).

#### Co-expression analysis

A gene with RPKM >0.5 in 50% of all developing cortex tissues was defined as a cortex-expressed gene. A total of 37 well-established IS-associated genes were considered a known IS gene set (Pavone et al., 2020). A total of 43 known genes associated with dominant epilepsy disorders, 33 genes related to neurodevelopmental disorders with epilepsy and 50 known genes associated with dominant EE syndromes were defined as a known EE gene set (Epi4K consortium and Epilepsy Phenome/Genome Project, 2017; Heyne et al., 2018). A total of 789 mono-allelic DD genes within definitive or strong evidence were from The Developmental Disorders Genotype-to-Phenotype database^4^ (Supplementary Table 2). Three gene sets were all applied to calculate the Spearman’s correlation coefficient with all cortex-expressed genes. Next, the mean of Spearman’s correlation coefficient of each cortex-expressed gene with known IS/EE/DD gene sets was computed. Percentiles of the average correlation coefficient between *MAST4* and known IS/EE/DD sets were acquired.

#### *MAST4* expression pattern in development brain

RNA-seq data at different developmental stages (from 8 postconceptional weeks to 40 years) for multiple brain areas were obtained from Brainspan^5^. RNA expression was normalized to reads per kilobase million (RPKM). Univariate linear regression for analyzing *MAST4* expression mode in CC and FC was achieved by lm() function in R^6^.

### Statistical methods

Comparison for CADD was performed using Wilcoxon test. Comparison for SIFT and Poly then was carried out using the Fisher test.

## Results

### Four individuals presented typical neurodevelopmental disorders with infantile spasm

**FIGURE 1:**
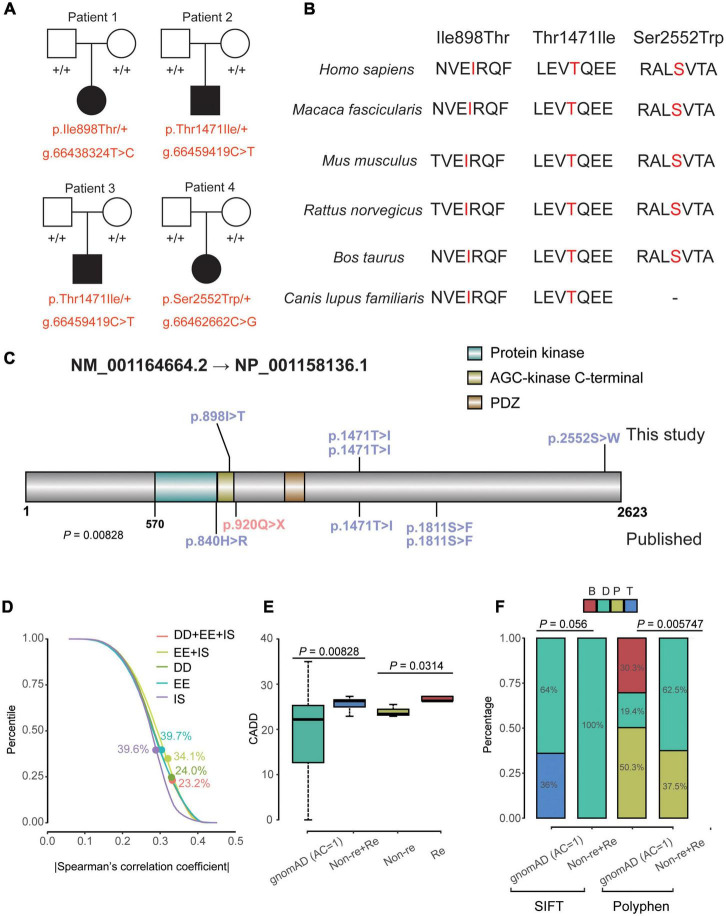
Clinical data and genetics of variants in *MAST4*. **(A)** Pedigree plot of the four unrelated families in this research. All affected probands (patient 1–4) are children of unaffected parents. Solid squares and solid circles represent affected males and females, respectively. Hollow squares and hollow circles represent unaffected males and females, respectively, +/+ represents a wild type and –/+ (red) represents a heterozygous genotype. **(B)** Comparison of conservative property of three *de novo MAST4* sites among multiple species. **(C)** Location of the variants in *MAST4*. Variants in blue and red represent missense and LGD events, respectively. Mutations on and under the *MAST4* belong to this study and published researches, respectively. **(D)** Percentile of |Spearman’s correlation coefficient| of the co-expression for all non-DD + EE + ISs genes vs. DD + EE + ISs genes, all non-DD vs. DD genes, all non- EE genes vs. EE genes and all non- ISs genes vs. ISs genes. Dots represent the percentile of *MAST4*. **(E,F)** Comparison of the CADD scores, SIFT bin and Polyphen bin between *de novo* missense variants (recurrent and non-recurrent) identified in Table 1 and the private missense variants identified in the non-neuro subset of gnomAD. AC, allele count; B, benign; CADD, Combined Annotation Dependent Depletion; D, deleterious; DD, developmental disorder; EE, epileptic encephalopathy; gnomAD, genome aggregation database; IS, infantile spasm; P, possibly damaging; SIFT, sortig intolerant from tolerant; T, Tolerant.

Firstly, we did not find Pathogenic and Like Pathogenic variation sites of genes related to NDD and epilepsy based on the trios’ exome sequencing. Then, we chose our candidate variant based on variant pathogenicity, conservation and hereditary mode. For the possible candidate variants, we further look into the gene functions which were related to neurodevelopment. Through all these methods to identify candidate variant, we finally chose MAST4 variants as candidates for our patients. Three *de novo* variants were identified in four unrelated patients (Figure 1). Clinical features of the individual with *MAST4* variants were summarized in Table 1.

**TABLE 1:** The clinical features of the affected individual with the *MAST4* variants.

Individual	Case 1	Case 2	Case 3	Case 4
Variant	c.2693T > C (p.I898T)	c.4412C > T (p.Thr1471Ile)	c.4412C > T (p.Thr1471Ile)	c.7655C > G (p.S2552W)
Variant type	Missense	Missense	Missense	Missense
Inheritance	*De novo*	*De novo*	*De novo*	*De novo*
ACMG Classification	Likely pathogenic	Uncertain significance	Uncertain significance	Likely pathogenic
Sex	Female	Male	Male	Female
Current age	1 y 7 m	2 y 5 m	2 y 3 m	3 y 3 m
Weight	12.4 kg	11 kg	10 kg	14 kg
Height	87 cm	88 cm	86 cm	100 cm
Head circumference	–	−2SD	−2SD	–
Age of onset	4 m	2 m	12 m	4 m
Gross motor delay	–	+	+	–
Speech	+	+	+	+
Cognitive delay/intellectual disability	+	+	+	+
Autistic features	Mildly delayed language development	Delayed motor and language development	Delayed motor and language development	Mildly delayed language development
Seizures	+	+	+	+
Epilepsy type	Infantile spasms	Infantile spasms	Infantile spasms	Infantile spasms
Epilepsy controlled	+	–	–	+
Brain MRI	Widened extra cerebral interspace of bilateral frontal and temporal lobe, dilatation of the bilateral lateral ventricles and dysgenesis of the corpus callosum	Bilateral ventriculomegaly, widened bilateral frontotemporal sulci and fissures; bilateral hippocampal MRS asymmetry, right NAA	Reduced white matter, dilatation of the bilateral lateral ventricles, delayed myelination of the posterior limb of the internal capsule and occipital lobe white matter	Sharp frontal anterior skull, decreased anteroposterior diameter of the skull
EEG	Multiple spike waves mixed with irregular slow waves and low amplitude fast waves with voltage decay	Widespread spike slow waves with low amplitude and the voltage decay	Generalized spike-slow waves following diffuse voltage decay	Poly spike-slow and irregular slow waves, partial hypsarrhythmia
Sleep disturbance	Unknown	+	+	Unknown
Facial dysmorphism	Normal	A wide nasi, gothic arch, penetrating palm	Penetrating palm, bulbous nose, low-set ears	Normal
Others	–	Congenital laryngeal cleft, trachea-bronchomalacia	Bilateral lateral ventricles, bilateral hydrocele of testes, congenital malformation of the toe, congenital hydronephrosis, undescended testes	–

**FIGURE 2:**
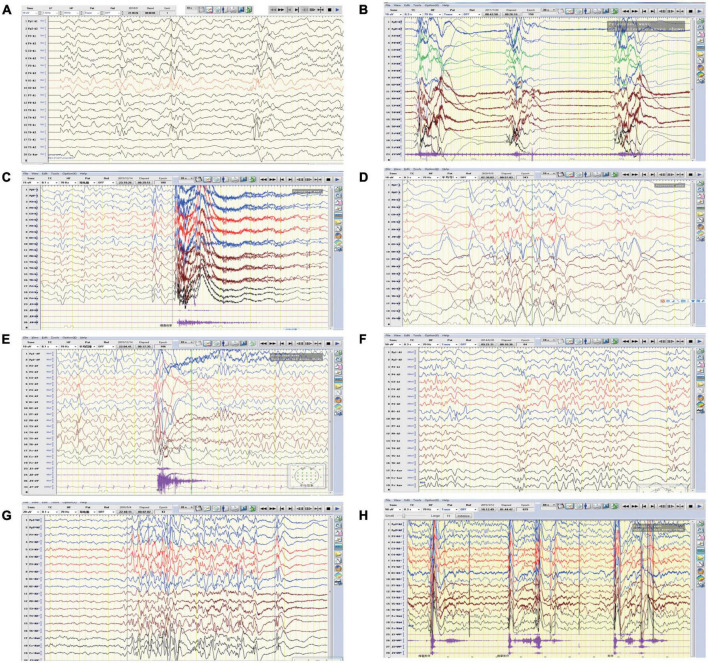
Representative EEGs of the affected individuals. **(A)** EEG of patient one revealed intermittent partial hypsarrhythmia (obtained at the age of 5 months). **(B)** EEG of patient one showed clusters of spastic seizures (at the age of 10 months). **(C,D)** EEG of patient two revealed convulsive episode and widespread spike slow waves with low amplitude at 4-month-old and 12-month-old, respectively. **(E)** EEG of patient three showed generalized spike-slow waves following diffuse voltage decay (at 15 months old). **(F–H)** EEG of patient four demonstrated poly spike-slow and irregular slow waves were more prominent on left, accompany with partial hypsarrhythmia and cluster of spasms (obtained at the age of 4 months and 12 months, respectively).

**FIGURE 3:**
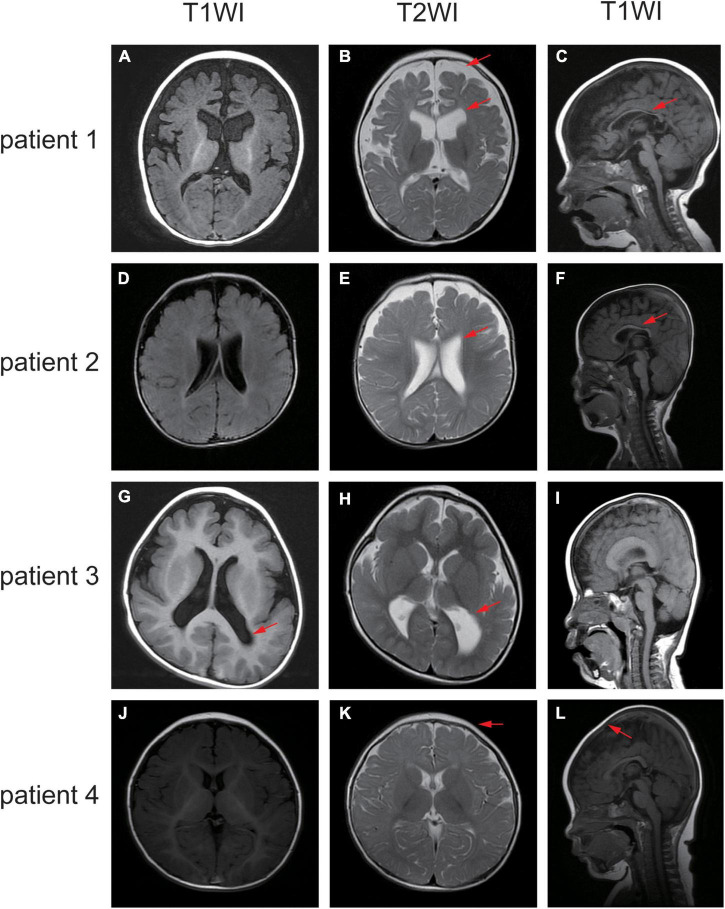
Different sequence of the brain MRI of the affected individuals. **(A–C)** MRI of patient one demonstrated widened extracerebral interspace, dilatation of the bilateral lateral ventricles and dysgenesis of the corpus callosum. **(D–F)** MRI of patient two showed bilateral ventriculomegaly and thin corpus callosum. **(G–I)** MRI of patient three revealed the posterior horn of bilateral ventricle and the trigone were enlarged bilaterally. **(J–L)** MRI of patient four elucidated sharp frontal anterior skull and decreased anteroposterior diameter of the skull. The red arrows indicate the positions of the lesions.

Patient 1 is a 1 year and 8 months old girl born to a healthy, non-consanguineous couple. At 2 months of age, the girl could not hold her head up steadily and lacked eye contact. Two months later, she started to present recurrent seizures, characterized by crying with abduction motions in bilateral upper limbs, flexion motions in lower limbs, body writhing toward right and both eyes gazing up and left. Cluster episodes of seizures occurred tens of seconds every time and two to three times a day. Convulsions of limbs occurred mainly at midnight after wake. At 5-month-old, the proband cannot lift her head steadily and presented hypertonia of the extremities. Her EEG mainly showed intermittent partial hypsarrhythmia at 5-month-old (Figure 2A) and clusters of spastic seizures: multiple spike waves mixed with irregular slow waves and low amplitude fast waves with voltage declining for 3–5 s (Figure 2B). Brain MRI showed widened extracerebral interspace of bilateral frontal and temporal lobe, dilatation of the bilateral lateral ventricles and dysgenesis of the corpus callosum (Figures 3A–C). At present, the 20-month-old girl had no cluster episodes of convulsions after the treatment with adrenocorticotropic hormone (ACTH) and her motor developmental milestone nearly reached normal.

Patient 2 is a boy, aged of 2 years and 6 months, who was born at 38 weeks *via* vaginal delivery due to oligohydramnios. At 2 months old, the infant was admitted to hospital because of intermittent convulsions and fever and the physical examination suggested microcephaly with a head circumference of 35 cm (<−2SD). His echocardiogram revealed patent foramen ovale (PFO) and tricuspid regurgitation. Brain MRI showed bilateral ventriculomegaly and widened bilateral frontotemporal sulci and fissures, suggesting brain developmental delay, bilateral hippocampal MRS asymmetry, and right low NAA indicating neuronal damage or loss in the hippocampal (Figures 3D–F). The onset EEG of the boy showed widespread spike slow waves with low amplitude and the voltage declined about 4.5 s (Figure 2C). Diffuse slow wave, spike-slow and slow wave were still seen after half a year (Figure 2D). Two months later, he was re-admitted due to recurrent convulsions manifested as facial flushing, cyanosis of lips, closed eyes, head pitched back, stiffness and shaking of limbs and foaming at the mouth. He was diagnosed with NDD, infantile spasm, trachea-bronchomalacia, cortical corpus callosum dysgenesis, and congenital laryngeal cleft. Seizures were poorly controlled with levetiracetam and topiramate. EEG at 1 year of age was still abnormal. At present, the patient was presented with delayed cognitive and language development.

Patient 3 is a 2-year-old male born after an uneventful delivery. The color ultrasound at 34 weeks of gestation showed widened bilateral ventricle. Shortly after birth, the child was diagnosed with dilatation of the bilateral lateral ventricles, tricuspid regurgitation, and bilateral hydrocele of the testes. The patient was hospitalized at 5 months of age because of head-up instability with a head circumference of 38.5 cm (<−2SD) and suboptimal limb activity. His developmental milestones were also severely delayed. He couldn’t raise his head until 7 months old, turn over until 10 months and still couldn’t crawl until now. Since the age of 3 months, he has been undergoing a continuous rehabilitation program including physical, speech and neuropsychology therapy. Brain MRI at 9 months old revealed reduced white matter, dilatation of the bilateral lateral ventricles, delayed myelination of the posterior limb of the internal capsule and occipital lobe white matter (Figures 3G–I). The boy had episodes of seizures at 1 year of age manifested as upturned eyes, trismus, and flexed twitching limbs. The onset EEG mainly showed generalized spike-slow waves following diffuse voltage decay suggesting infantile spasm (Figure 2E).

Patient 4 is a 3-year-old female who was born at full term from a normal pregnancy. She couldn’t make sounds and head up unsteadily until 4 months of age. Shortly after, the girl developed seizures characterized by dozen times of nodding and body flexing, 5–6 min in each episode. The EEG showed poly spike-slow and irregular slow waves were more prominent on the left, accompany by partial hypsarrhythmia at 4 months old (Figures 2F, G) and a cluster of spasms at 1 year old (Figure 2H). Brain MRI at 11 months old showed a sharp frontal anterior skull and decreased anteroposterior diameter of the skull (Figures 3J–L). Then the girl was diagnosed with infantile spasm and her seizures were well controlled with topiramate monotherapy. At present, the girl had reached a mildly delayed motor development combined with mildly delayed behavioral development.

### *In silico* parameters prompt *de novo MAST4* variants are pathogenic especially its recurrent sites

Although clinical evidence has been presented to implicate *MAST4* an EE/DD risk gene, its pathogenicity needs further elucidation based on the characteristics of *MAST4* and variants on its protein.

For gene, constraint’s parameters of *MAST4* (Missense Z-score = 1.93 and RVIS% = 8.35) suggested that *MAST4* is moderately intolerant for missense variants. Statistical analyses of publically available data on co-expression revealed *MAST4* ranks higher concerning the correlations with known DD genes (Percentile = 24.0%) compared with well-established IS/EE genes (IS: Percentile = 39.6%; EE: Percentile = 39.7%) (Figure 1D and Supplementary Table 3).

**FIGURE 4:**
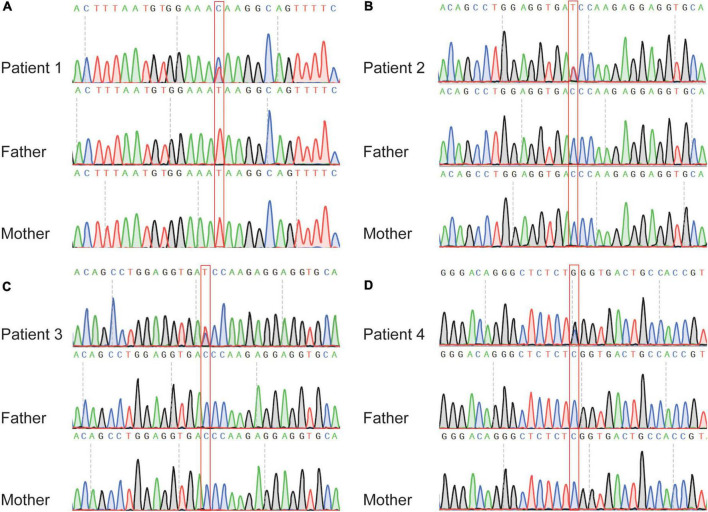
Sanger sequencing of the affected individuals with *MAST4* variants identified in this study. **(A)** c.2693T > C in patient one trio. **(B)** c.4412C > T in patient two trio. **(C)** c.4412C > T in patient three trio. **(D)** c.7655C > G in patient four trio. The red boxes denote the positions of the variants.

For variants on it, all three variants are not found in human population databases gnomAD, ExAC, and 1,000 genomes (non-neuro subset) (Table 2). These three heterozygote missense variants were identified in four unrelated cases as follows: chr5:66438324 (c.2693T > C: p.Ile898Thr), chr5:66459419 (c.4412C > T: p.Thr1471Ile) and chr5:66462662 (c.7655C > G:p.Ser2552Trp). All of them are *de novo* variants, where in bidirectional Sanger sequencing confirmed such findings (Figures 1A, 4). All three variants are predicted to be damaging or probably damaging through multiple different bioinformatics software (Table 2) and the affected amino acid is highly conserved across multiple species (Figure 1B). The missense variant p.Ile898Thr is mapped to the AGC-kinase C-terminal, which can modify other proteins by adding a phosphate group (Figure 1C). And the other variant p.Ser2552Trp is located in a phosphoserine-modified residue which may affect cell membrane stability and signal transduction (Uniport, O15021). The last variant p.Thr1471Ile was screened out in two unrelated patients and is located in the domain with the function unclear yet.

**TABLE 2:** Summary of *MAST4 de novo* missense variants identified in neurodevelopmental disorders.

Index	1	2	3	4	5	6	7	8	9
Sample ID	95,715	Case 1	DDD13k.08536	DDD13k.00269	Case 2	Case 3	18,847	66,810	Case 4
gDNA change (chr5,hg19)	g.66437967A > G	g.66438324T > C	g.66440524C > T	g.66459419C > T	g.66459419C > T	g.66459419C > T	g.66460439C > T	g.66460439C > T	g.66462662C > G
Function	Missense	Missense	Stop gain	Missense	Missense	Missense	Missense	Missense	Missense
Coding change	c.2519A > G	c.2693T > C	c.2758C > T	c.4412C > T	c.4412C > T	c.4412C > T	c.5432C > T	c.5432C > T	c.7655C > G
Protein change	p.840H > R	p.898I > T	p.920Q > X	p.1471T > I	p.1471T > I	p.1471T > I	p.1811S > F	p.1811S > F	p.2552S > W
SIFT	D	D		D	D	D	D	D	D
Polyphen	D	D		P	P	P	D	D	D
CADD	25.5	22.9	38	26.3	26.3	26.3	27.3	27.3	23.4
MutationTaster	D	D	A	D	D	D	D	D	D
PROVEAN	D	D		D	D	D	D	D	N
M-CAP	D	D		D	D	D	D	D	D
VEST4	0.944	0.715	0.36	0.719	0.719	0.719	0.965	0.965	0.512
GERP	5.42	5.78	5.16	5.4	5.4	5.4	5.14	5.14	5.06
phyloP	1.312	1.138	1.026	1.026	1.026	1.026	1.026	1.026	0.08
**ExAC**									
**1,000 genomes**									
gnomAD	0.0000112								
Inheritance	*De novo*	*De novo*	*De novo*	*De novo*	*De novo*	*De novo*	*De novo*	*De novo*	*De novo*
PMID	33057194	This study	33057194	33057194	This study	This study	33057194	33057194	This study
Cohort Size	31058	Unknown	31058	31058	Unknown	Unknown	31058	31058	Unknown
Primary diagnosis	DD	IS	DD	DD	IS	IS	DD	DD	IS

Isoform: NM_001164664.1; A, disease causing automatic; DD, developmental disorder; IS, infantile spasm; P, possibly damaging; D, deleterious; N, neutral.

After we integrated IS/EE/DD studies with large GS/ES cohorts from Gene4Denovo, eight *de novo* missense variants and one nonsense mutation were obtained (Table 2 and Figure 1C). Probands carrying *MAST4* variants from published research had DD symptoms, which implied that *de novo MAST4* variants may mainly contribute to DD phenotypes combined with previous results in Figure 1D and Supplementary Table 3. Furthermore, we chose three representative constraint’s metrics of missense variants for follow-up analysis, Combined Annotation Dependent Depletion (CADD), Sorting Intolerant From Tolerant (SIFT) and Polyphen. Compared to private *MAST4* missense mutations in gnomAD non-neuro subset, higher CADD scores were found in *de novo* missense variants of integrated IS/EE/DD cohorts (*P* = 0.00828) (Figure 1E). Similarly, integrated *de novo* missense events had more pathogenic components predicted by SIFT and Polyphen in contrast to private missense variants in gnomAD non-neuro subset (SITF: *P* = 0.056; Polyphen: *P* = 0.005747), even if *P*-value in SIFT was close to significance (Figure 1F). Strikingly, two recurrent sites, p.Thr1471Ile and p.Ser1811Glu, were carried by three (two from our study and one from published research) and two (all from published research) unrelated probands, separately (Table 2 and Figure 1C). We noticed higher CADD scores from two recurrent events than those in non-recurrent mutations from integrated IS/EE/DD cohorts (*P* = 0.0314), which indicated more deleteriousness of recurrent variants in *MAST4* (Figure 1E).

### Spatio-temporal modes of *MAST4*

**FIGURE 5:**
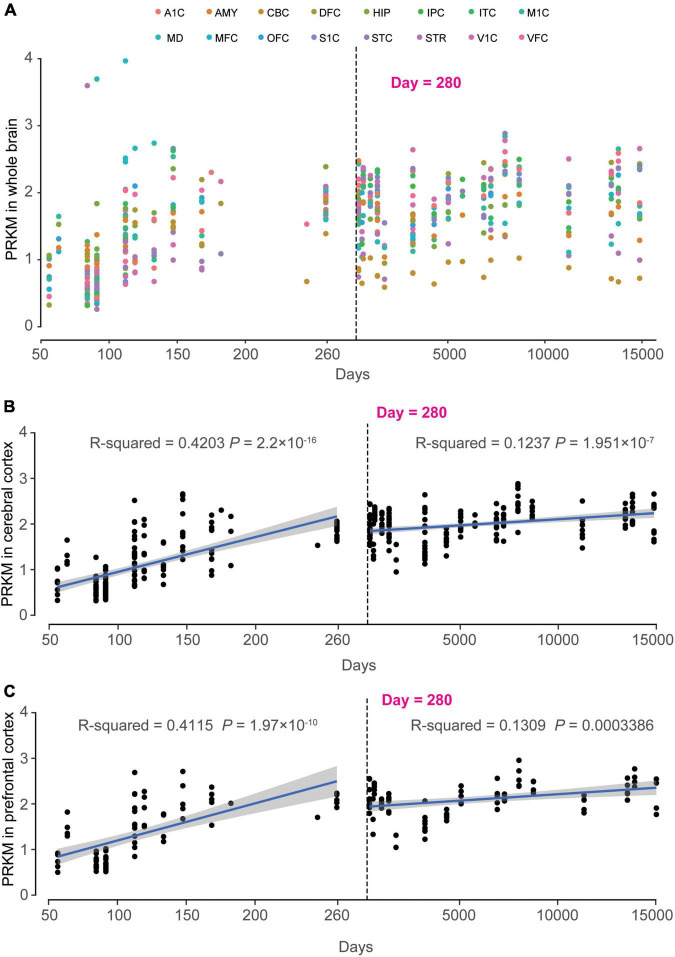
Dynamic expression pattern of *MAST4* in human development brains. The x-axis is the age of samples in days and y-axis is the RPKM of *MAST4*. The dashed line indicates the birth day. **(A)** Expression of *MAST4* during the whole life in different brain regions. A1C, primary auditory cortex; AMY, amygdaloid complex; CBC, cerebellar cortex; DFC, dorsolateral prefrontal cortex; HIP, hippocampus; IPC, inferior parietal cortex; ITC, inferolateral temporal cortex; M1C, primary motor cortex; MD, mediodorsal nucleus of thalamus; MFC, medial prefrontal cortex; OFC, orbital frontal cortex; S1C, primary somatosensory cortex; STC, superior temporal cortex; STR, striatum; V1C, primary visual cortex; VFC, ventrolateral prefrontal cortex. **(B,C)** Expression of *MAST4* in the cerebral cortex and prefrontal cortex regions of human brain, respectively. Univariate regression analysis was applied in prenatal brains (left) and postnatal brains (right), separately. Blue line represents the regression line and the gray region indicates 95% confidence interval. The text shows the *R*^2^ and *P*-value of regression.

In order to clarify the important role of *MAST4* in brain development, the RNA sequencing data from BrainSpan was applied to find out the spatiotemporal expression pattern of *MAST4*. We found a time-dependent up-regulation during 50–150th days *in utero* for *MAST4* (Figure 5A). Differently, most brain regions retained the high expression of *MAST4* during whole post-birth periods (Figure 5A). To reveal a dynamic change of *MAST4* in IS/EE/DD relevant brain regions during periods of brain development, like cerebral cortex (CC) and prefrontal cortex (FC) (Treiman, 2001; Hsu and Jaeggi, 2014; Severino et al., 2020; Galovic et al., 2021), a univariate linear regression analysis was performed. In CC and FC, expression of *MAST4* was more significantly up-regulated during the period of embryonic development (CC: *R*^2^ = 0.4203 and *P* = 2.2 × 10^–16^. FC: *R*^2^ = 0.4115 and *P* = 1.97 × 10^–10^) than that during the postnatal period (CC: *R*^2^ = 0.1237 and *P* = 1.951 × 10^–7^. FC: *R*^2^ = 0.1309 and *P* = 0.0003386) (Figures 5B, C). These data implicated a potential role of *MAST4* during embryonic developing periods.

## Discussion

In the present study, we described three *de novo* heterozygous missense *MAST4* variants in four unrelated patients with IS and varying levels of developmental delays. The detail variations were identified as follows: 5:66438324-T-C (p.Ile898Thr), 5:66459419-C-T (p.Thr1471Ile) and 5:66462662-C-G (p.Ser2552Trp). The variant p.Ile898Thr located in the AGC-kinase C-terminal domain and p.Ser2552Trp was right in a phosphoserine-modified residue which may affect cell membrane stability and signal transduction. The variant p.Thr1471Ile was identified in two unrelated individuals with severe developmental retardation, indicating a greater probability of function-altering and pathogenic hotspot variants. According to a previous study reanalyzing large-scale whole exome sequencing data, *MAST4* was identified as a novel gene significantly associated with developmental disorders (Kaplanis et al., 2020). Intriguingly, coupled with our findings, two recurrent missense variants, 5-66460439-C-T and 5-66459419-C-T were identified, which greatly improved the possibility of pathogenicity. The results of the bioinformatic analysis tend to elucidate that *de novo MAST4* variants may contribute to DD phenotypes compared to IS combined with previous report. However, this might because *MAST4* gene variations are rarely reported and the patients may also have seizures in the cohort with DD. Our study presented evidence that *MAST4* could be a candidate NDD with IS gene and therefore expanded the genetic and phenotypic spectrum.

*MAST4* belongs to the microtubule-associated serine/threonine kinase (*MAST*) family and is widely expressed in the nervous system (Garland et al., 2008). *MAST4* protein distribution in the central nervous system is predominant in cerebellar Purkinje cells, hippocampus and white matter-containing regions (Garland et al., 2008) and its expression was increased in the mouse hippocampus following seizure activity (French et al., 2001). At present, there have been few basic studies on *MAST4* in the nervous system. These results suggest that *MAST4* variants disrupt cells’ self-renewal, proliferation, and differentiation. Gongol et al. (2017) constructed a missense mutant plasmid (E682A) and the detection of kinase activity *in vitro* suggested that the missense variant disrupts kinase activity. We also constructed the *MAST4* wild type and three corresponding mutant plasmids. However, the plasmids were too long to be transfected into cells, and neither was the lentivirus.

Recent studies have shown close relationships between NDD and variants in the *MAST* family members. *MAST1* was the first family member found to be linked with neurological conditions (McMichael et al., 2015). And it appeared to play a vital role in interacting with the dystrophin/utrophin-associated complex located in the neuromuscular junction and postsynaptic region of the central synapses (Lumeng et al., 1999). Previous studies suggested that *MAST1* was a novel candidate gene associated with multiple neurological diseases, including mega-corpus-callosum syndrome, cerebral palsy, developmental delay and intellectual disability (McMichael et al., 2015; Tripathy et al., 2018; Ben-Mahmoud et al., 2020; Rodríguez-García et al., 2020). These observations suggest that *MAST1* is crucial in neuronal development and may be a new potential biomarker for neuronal developmental disorders. Mast 3, as well as Mast 1, another protein of this family, has been previously reported to be involved in neurological disease. Iwama et al. (2019) firstly showed that *de novo* variants in the STK domain of the *MAST3* gene resulted in Rett syndrome-like phenotype. Subsequently, two studies illustrated the role of *MAST3* variants in neurodevelopmental delay and developmental epileptic encephalopathy, respectively (Shu et al., 2021; Spinelli et al., 2021). Morange et al. (2021) proposed that *MAST2* variants may cause venous thromboembolism by interfering with the hemostatic balance of endothelial cells. Taken together, our analysis firstly reported the variations in *MAST4* associated with NDD and IS and took a step further indicating the *MAST* family members play an essential role in neurodevelopment.

Combining the findings of genetic and bioinformatic analysis, our findings may confirm *MAST4* as a novel gene for NDD with IS. However, there are some limitations in this study to be mentioned. The variants were mainly identified in in-person visiting patients with seizures and the lack of an observable phenotype in other systems does not rule out the possibility that other undiscovered biological and clinical phenotypes exist. Furthermore, more cases and functional investigations are necessary to demonstrate robust genotype-phenotype correlations and the underlying mechanisms.

## Conclusion

Here, we identified three heterozygous missense *MAST4* variants in four unrelated patients with a spectrum of IS and varying developmental delays. Our findings evaluate the genotype-phenotype correlations of MAST4 gene in NDDs and IS, which expands the genetic landscape and future genetic counseling of NDDs with IS.

## Data availability statement

The datasets that support this article are available on request from the corresponding authors. The data are not publicly available due to concerns regarding privacy and participant/patient anonymity.

## Ethics statement

The studies involving human participants were reviewed and approved by the Ethics Committee of Maternal and Child Health Hospital of Hunan Province (2020-S003). Written informed consent to participate in this study was provided by the participants’ legal guardian/next of kin. Written informed consent was obtained from the minor(s)’ legal guardian/next of kin for the publication of any potentially identifiable images or data included in this article.

## Author contributions

JY and HW: conceptualization and methodology. XZ and NX: data curation and writing—original draft preparation. YC, YLC, PW, and WG: collection and evaluation of the clinical and genetic evidence. AL and YP: data analysis. BX and LS: writing—reviewing and editing. All authors read and approval of the final manuscript.

## Footnotes

1
http://broadinstitute.github.io/picard/

2
www.pubvar.com

3
http://www.genemed.tech/gene4denovo/home

4
www.deciphergenomics.org/ddd/ddgenes

5
http://www.brainspan.org/

6
www.r-project.org/

## Supplementary material

Supplementary Table 1 The relationships of family 1–4 in this study. Kinship: Estimated kinship coefficient from the SNP data. >0.354, (0.177, 0.354), (0.0884, 0.177) and (0.0442, 0.0884) corresponds to duplicate/MZ twin, 1st-degree, 2nd-degree, and 3rd-degree relationships respectively.

Supplementary Table 2 Well-established known IS/EE/DD gene sets.

Supplementary Table 3 Percentile of Spearman’s correlation coefficient| of the co-expression for all non-DD + EE + ISs genes vs. DD + EE + ISs genes, all non-DD vs. DD genes, all non- EE genes vs. EE genes and all non- ISs genes vs. ISs genes.
